# Identification and characterization of satellite DNAs in *Poa* L.

**DOI:** 10.1186/s13039-020-00518-x

**Published:** 2020-11-16

**Authors:** Linna Wei, Bo Liu, Chunping Zhang, Yang Yu, Xiaoxia Yang, Quanwen Dou, Quanmin Dong

**Affiliations:** 1 State Key Laboratory of Plateau Ecology and Agriculture in the Three River Head Waters Region, Qinghai Academy of Animal and Veterinary Science, Xining, 810001 Qinghai Province China; 2 Qinghai Provincial Key Laboratory of Adaptive Management on Alpine Grassland, Qinghai University, No. 1 of Weier Road in Shengwuyuan District of Xining City, Xining, 810016 Qinghai Province China; 3 Key Laboratory of Crop Molecular Breeding Qinghai Province, Northwest Institute of Plateau Biology, The Chinese Academy of Sciences, Xining, 810008 Qinghai Province China

**Keywords:** *Poa*, Satellite DNA, Fluorescent in situ hybridization, FISH

## Abstract

**Background:**

*Poa* L. is a large genus of grass in Gramineae, among which *P. pratensis* is widely cultivated as turf and forage. Satellite DNA is the main components of the plant genome. Information of satellites will helpful for dissection the genome composition and definition of the phylogeny relationship of these species. However, the knowledge about the satellites in genus *Poa* is still limited.

**Results:**

Four satellite DNAs were identified using the Repeat Explorer pipeline in HiSeq Illumina reads from diploid plants in *P. malaca* (2n = 26). Two satellites showed high similarity with the previously identified *Pp*Tr-1 and *Pp*Tr-3, whereas two others are newly identified with the monomer of 326 bp (*Poa*-326) and 353 bp (*Poa*-353) respectively. The clone DNAs of *PpTr*-1 and *PpTr*-3, and oligonucleotides designed representing satellites Poa-326 and Poa-353 were probed to test on chromosomes across 13 *Poa* speceis with different polyploidy level by fluorescent in situ hybridization (FISH). *Pp*Tr-1, *Pp*Tr-3, and *Poa*-362 were stably positioned in the subtelomeric regions in nearly all species with the variation of hybridization sites number. However, *Poa*-353 showed different FISH patterns of multiple regions with the variation of hybridization intensity and distribution sites across species. In addition, 5S rDNA and 45S rDNA were used to characterize the genome of the *Poa* species. Four rDNA FISH patterns were revealed in the tested species.

**Conclusion:**

Four identified satellite were high conservable across *Poa* species. Genome distribution of these satellites can be characterized by FISH. The variation of satellite DNAs and rDNA chromosomal distributions between species provide useful information for phylogenetic analysis in genus *Poa*.

## Background

The genome of high plants contains much of repetitive sequences [1–3] most of which are genome dispersed transposable elements (DNA transposons and retrotransposons), and tandem arranged satellite DNA [4]. Satellite DNAs are mostly species and genus specific, and usually most variable in abundance and chromosomal distribution between species [5–7]. Satellite DNA is majorly distributed in chromosomal heterochromatic regions, and regarded in functions in chromosomal structure maintenance, centromere formation, homologue chromosome recognition, and even gene function adjustment [8–10]. Abundance and distribution of satellite DNA in chromosomes can be detected by a technique of Fluorescence in situ hybridization (FISH). Satellite DNA-based chromosomal markers are particularly useful for chromosome identification and for karyotype evolution analysis.

*Poa* L. is a large genus of grass, including up to 500 species mainly in temperate and arctic zones thought the world [11, 12]. The center of diversity of *Poa* is considered to be Eurasia [11]. About 100 species of *Poa* are described in China [13]. *P. pratensis* (Kentucky bluegrass) is a most useful *Poa* species, which is used worldwide as a temperate turf grass and forage crop. *P. pratensis* var. *anceps* and *P. crymophila* are utilized as ecology restoration plants and forage crops in Qinghai-Tibet plateau, China [14]. *Poa* species presents high variability with a wide range of chromosome numbers, due to high variable polyploidy, interspecific hybridization and facultative apomixis [11, 12, 15–17]. Characterization of the genome composition and definition of the phylogeny relationship of these species is still challenging.

Four satellite DNAs were isolated from *P. pratensis* by construction and screening cot-1 libraries, and the conservation and variability of these satellites was tested in only one more related *Poa* species [18]. It is still unknown the phylogenetic distribution of these satellite DNAs in other more *Poa* species. Furthermore, *P. pratensis*, as a facultative apomixes species, presents high polyploidy with large genomes. It is possible that the other more satellite DNAs are missed with a conventional method by screening limited libraries clones. The developed RepeatExplorer, a graph-based sequence clustering program, is powerful to identify various types of repetitive DNA elements in de novo by using a set of genomic sequences produced by the next generation sequencing technique (NGS) [19, 20]. It is possible to get the most repetitive sequences even using low-coverage sequencing data. A combination of RepeatExplorer and FISH has become a popular methodology to identify and characterize major satellite repeats in many plant species [21–23].

In this study, satellite DNAs was identified using low-coverage genomic DNA sequence data from the next-generation sequencing platform in a diploid *Poa* species. In addition, chromosomal distribution of these satellites was characterized in more than 10 different *Poa* species including *P. pratensis* by FISH. Finally, the phylogenetic significance of the satellite DNA across *Poa* species was discussed.

## Materials and methods

### Plant materials

Total 13 *Poa* L. species were used in this study. They are *P. malaca* Keng ex P. C. Kuo, *P. elanata* Keng ex Tzvel., *P. megalothysa* Keng ex Tzvel., *P. poophagorum* Bor., *P. sphondylodes* Trin., *Poa paucifolia* Keng ex L. Liou, *Poa orinosa* Keng ex P. C. Kuo, *P. sinoglauca* Ohwi, *P. crymophila* Keng ex C. Ling, *P. subfastigiana* Trin., *P. pratensis* Var. anceps, *P. pratensis* L. Four cultivars, named *P. pratensis* ‘Qinghai’, ‘Park’, ‘Geronimo’, and ‘Sapphire’ were contained in species *P. pratensis*. All above plant materials, except *P. pratensis* cultivars Park, Geronimo, and Sapphire, are sourced in Qinghai-Tibet plateau, China. Seeds of cultivars ‘Park’, ‘Geronimo’, and ‘Sapphire’ were all sourced from USA.

### High-throughput sequencing of genomic DNA

Total genomic DNA of the plants was isolated from your leaves. Paired-end sequencing (2 × 125 bp) of total genomic DNA was performed using Illumina HiSeq 2000 (Illumina, Inc.) at Benagen company.

### Clustering of satellite DNAs

The obtained NGS reads were upload to Repeat Explorer (https://repeatexplorer.org/) for a graph-based sequence clustering using a pipeline procedure [19, 20]. The putative satellite repeats were predicted based on their unique graphic characteristics.

### Chromosome preparation

Metaphase chromosomes of root tip cells were used for FISH analysis. A detailed chromosome preparation was according to Zhao et al. [18].

### FISH procedure

Two kinds of labelling methods, random primer labelling and end labelling, were adopted for probe preparation. The 5S rDNA was amplified by PCR using genomic DNA of *P. pratensis* according to Fukui et al. [24]. The satellite DNA of *Pp*Tr-1 and *Pp*Tr-3 were amplified from the previous made clones [18]. The 5S rDNA, *Pp*Tr-1 and *Pp*Tr-3 were by a random primer labelling method, described by Dou et al. [25]. The 45S rDNA was represented by end labelled oligonucleotide pTa71-1 and pTa71-2 described by Tang et al. [26]. Two other oligonucleotide designed to represent the newly identified satellite DNAs were end-labelled with FAM (green) or TAMRA (red). The FISH hybridization procedure and micrometry followed Zhao et al. with minor modification [18]. Before hybridization reaction, the hybridization mixture with oligonucleotide probes was placed directly onto the denatured slide preparation, rather than the hybridization mixture with the ddsDNA probe was denatured in boiling water for 5 min.

## Results

### Identification and characterization of satellite DNAs in a diploid of *P. malaca*

Primary screening of the chromosome number showed that the varied polyploids were existed in the tested *Poa* species. Specially, the diploidy form was identified in the population of *P. malaca*. Taking think that the diploid form contains less genome content than the tetraploid or other high polyploidy, we selected the diploid form of *P. malaca* for the candidate for high-throughput sequencing. The Illumina HiSeq data (genome coverage c. 30.3%) from diploid form of *P. malaca* was applied to the RepeatExplorer pipeline clustering tool. Four putative satellite DNAs were identified with the monomer 365 bp, 189 bp, 326 bp, and 353 bp from the output graphs. The satellite 365 bp has the most abundance about 0.85% of the genome, while the 189 bp, 326 bp, and 353 bp satellite has about 0.52%, 0.40%, and 0.24% respectively. Additionally, to test the similarity between the identified satellites with other repetitive sequences, nucleotide BLAS was conducted in the gene bank of NCBI. (https://www.ncbi.nlm.nih.gov/). The results showed that the 365 bp satellite has a high similarity with the tandem repeat *Pp*Tr*-1* identified in *P. pratensis* (KY618838.1) with an identity of 98.8% and 100% coverage, and the 189 bp satellite has a high similarity with the tandem repeat *Pp*Tr*-*2 and *Pp*Tr*-*3 in *P. pratensis* (KY618841.1 and KY618840.1) with the identity of 98.9% and 100% coverage both. However, no significant similarity was found for both 326 bp and 353 bp satellite. Thus, we thought that the 326 bp and 353 bp satellites are two novel satellite DNAs identified in *Poa*, and we named them as *Poa*-362 and *Poa*-353.

**Fig. 1:**
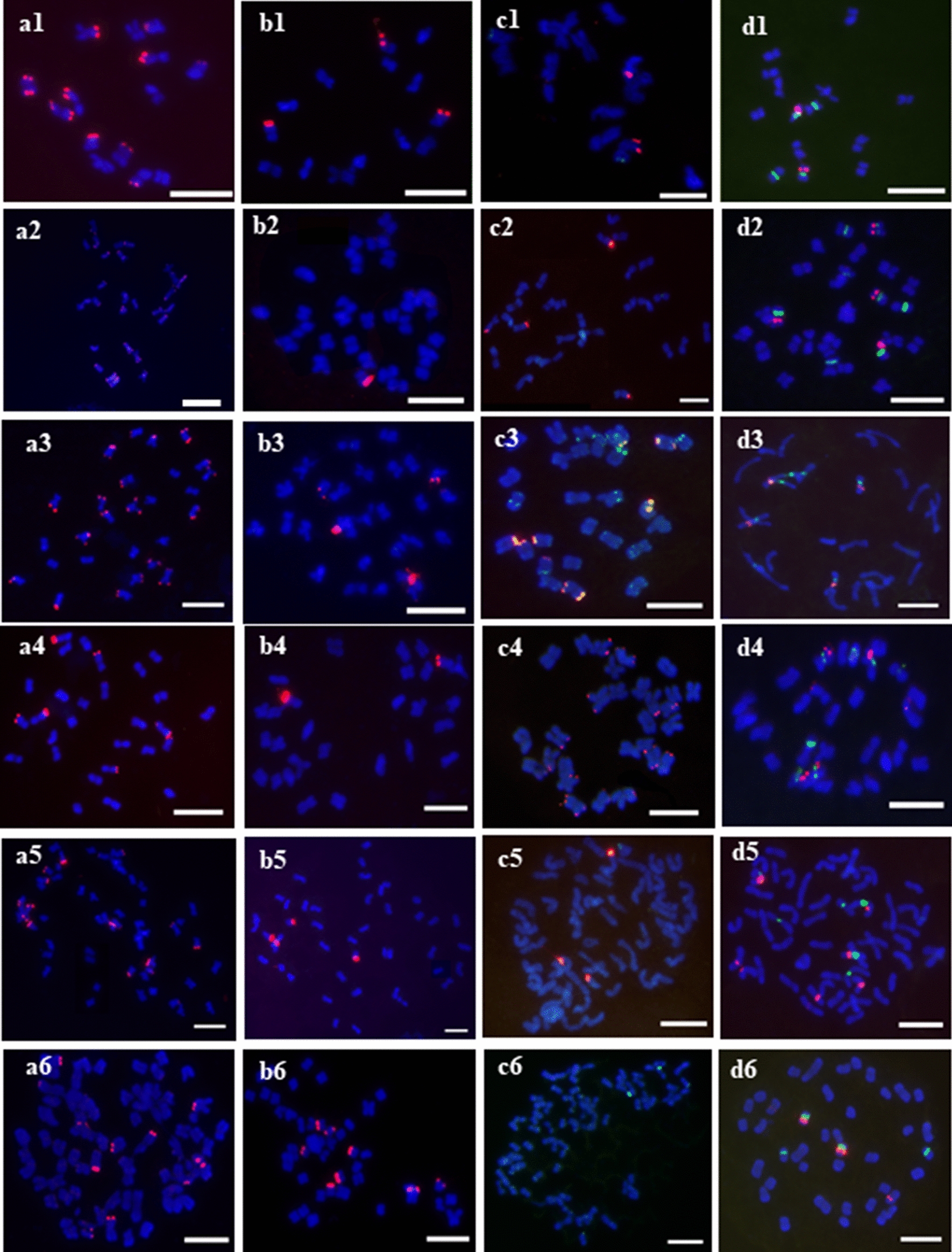
The mitotic metaphase FISH patterns in representative species: **a**–**d** were probed by *Pp*Tr-1 (red), *Pp*Tr-2 (red), *Poa*-362 (red) plus *Poa*-353 (green), and 5S rDNA (red) plus 45S rDNA (green) respectively. 1, 2, 3, 4, 5 and 6 were *P. malaca* (2n = 14), *P. malaca* (2n = 28), *P. sphondylodes* (2n = 28), *P. elanata* (2n = 28), *P. subfastigiana* (2n = 52–56), and *P. pratensis* ‘Sapphire’ (2n = 44–53). (bar = 10 μm)

Examination of chromosomal distribution of the four above satellites carried out in the diploid form of *P. malaca* with the chromosome number of 14*.* Since the satellite 365 bp and 189 bp showed the high similarity with the previous identified tandem repeats*Pp*Tr-1, *Pp*Tr-2 and *Pp*Tr-3 in *P. pratensis*, the clone sequence probes *Pp*Tr-1 and *Pp*Tr-3 [18] were used for representing the satellite 365 bp and 189 respectively in this study. Oligonucleotide FISH probes were designed for *Poa*-362 and *Poa*-353 from the sequences of the monomers using DNAman software package (Lynnon Biosoft, Quebec, Canada) (Table 1). The FISH patterns showed that *Pp*Tr-1, *Pp*Tr-3, and *Poa*-362 produced high intensity signals of 12, 3, and 2 respectively, which are all located in the terminal positions of the chromosomes. Relatively, *Poa*-353 produced less and weak hybridization signals of 2–3 in the subtelomeric and intercalary regions (Fig. 1a1–d1). The opulence of the four satellites detected by FISH is appropriate to the abundance of those derived from the high-throughput data analysis in some extent.

**Table 1:** Oligonucleotide FISH probes

Satellite DNA	Probe	Sequence (5′–3′) and flourochrome label	Amount applied to preparation (ng)
*Poa*-362	*Poa*-362-1	TAMRA-GTTAGGTCTCGCCACACTTG	10 ng each mixture
	*Poa*-362-2	TAMRA-GACGAAATGTAATGTCTCCTGG	
*Poa*-353	*Poa*-353-1	FAM-CCCACAACCTACCAACACAT	10 ng each mixture
	*Poa*-353-2	FAM-CCACAACCTACCAACACATTC	
	*Poa*-353-3	FAM-CAACCTAACACAACCAAGCA	
	*Poa*-353-4	FAM-AGTTCCGACACTTAGAGGTGA	

### Characterization of satellite DNA across different *Poa* species

In total, 12 different *Poa* species are involved in this study. Additionally, *P. pratensis* contains one subspecies *P. pratensis* Var. *anceps*, and four *P. pratensis* cultivars ‘Qinghai’, ‘Park’, ‘Geronimo’, and ‘Sapphire’. The investigated species appeared different ploidy levels with one diploidy form, 10 tetraploid species with 28 chromosomes, and 2 high polyploidy species (*P. subfastigiana* and *P. pratensis*) with a variable chromosome number (Table 2). All the probes *Pp*Tr-1, *Pp*Tr-3, and *Poa*-362 produced hybridization signals in the subtelomeric regions across all the investigated positive species (Fig. 1a–c). However, hybridizations of *Poa*-353 were detected in subtelomeric, intercalary, and pericentric regions in the investigated samples (Fig. 1c). It suggests that the genomic distribution pattern of *Poa*-353 is totally different from those of the other three satellites in genus *Poa*.

**Table 2:** Chromosomal distribution of the repetitive sequences across the *Poa speceis*

Species	Total Chr. number	Hybridization sites number					
		PpTR-1 (terminal)	PpTR-3 (terminal)	Poa-362 (terminal)	Poa-353 (multiple)	5S rDNA	45S rDNA
*P. malaca*	14	12	3–4	2	2–3	2(2) + 0	2(2) + 2(2)
	28	22	2	4	4	4(4) + 0	4(4) + 4(4)
*P. megalothysa*	28	8–11	2	10	11	4(4) + 0	4(4) + 4(4)
*P. poophagorum*	28	12–13	1–2	12	6	4(4) + 0	4(4) + 4(4)
*P. sphondylodes*	28	26	4–6	10	20	4(4) + 0	4(4) + 4(4)
*P. pagophila*	28	6–7	1	5–6	8	4(4) + 0	4(4) + 4(4)
*P. sinoglauca*	28	24–26	1	16	2	4(4) + 0	4(4) + 4(4)
*P. orinosa*	28	22–23	0	11	10	4(4) + 0	4(4) + 4(4)
*P. elanata*	28	8	1–2	17–18	0	4(4) + 2(2)	4(4) + 2(2)
*P. paucifolia*	28	7–8	0	24	12	4(4) + 0	4(4) + 2(2)
*P. crymophila*	28	12	1	7–8	4	4(4) + 0	4(4) + 2(2)
*P. subfastigiana*	52–56	16–19	5–6	2–3	5–6	3(3) + 5(5)	3(3) + 4(4)
*P. pratensis* Var. anceps	38–42	6–8	2–4	2	30	2(2) + 4(4)	2(2) + 3(3)
*P.pratensis* ‘Qinghai’	42–45	12–15	2	1–3	23–28	2(2) + 4(4)	2(2) + 6(6)
*P.pratensis* ‘Park’	48–50	15–17	9–11	0–1	16–18	3(3) + 1(1)	3(3) + 2(2)
*P.pratensis* ‘ Geronimo’	53–54	11–12	16–17	0	28–33	2(2) + 2(2)	2(2) + 4(4)
*P.pratensis* ‘Sapphire’	44–53	10–12	8–10	0	20–30	2(2) + 2(2)	2(2) + 2(2)

A formula a(b) + c(d) was used to described the rDNA pattern. a indicates sites number of 5S rNA or 45SrDNA which shared a common chromosome each other; b indicates number of chromosomes carrying both 5S rNA and 45SrDNA; c indicates sites number of 5S rNA or 45SrDNA which are solely located; c indicates number of chromosomes carrying solely 5S rNA or 45SrDNA

The number of hybridization sites between different satellites is varied. About 14, 4, 8, and 12 hybridizations were detected by *Pp*Tr-1, *Pp*Tr-3, *Poa*-362, and *Poa*-353 respectively in an average in the total investigated samples, although varied hybridization numbers were observed in the different species. The *Pp*Tr-1 presents the intense hybridization in each species, and high sites number varied from 6 to 26 (Fig. 1a, Table 2). In the tetraploid *Poa* species, the *Pp*Tr-1 produced about 15 hybridization sites averagely, with more than 20 hybridization sites in *P. malaca*, *P. sinoglauca* and *P. orinosa*. In the high polyploidy species, the *Pp*Tr-1 probed about 12 hybridization sites averagely (Table 2). The *Pp*Tr-3 produced intense hybridizations in all species except in *P. orinosa* and *P. paucifolia*. The *Pp*Tr-3 probed about 1.5 hybridization sites in tetraploid species, whereas about 7 in high polyploidy species averagely. The hybridization sites of *Poa*-362 were detected in all tested species except in *P. pratensis* cultivar ‘Geronimo’ and ‘Sapphire’ (Table 2). The average of 12 hybridization sites of *Poa*-362 was probed in tetraploid species with an exceptional number of 24 in *P. paucifolia*, while about 1 site was detected in the high polyploidy species averagely. The hybridization sites of *Poa*-353 were detected in all tested species except in *P. elanata* (Table 2). The average of 12 hybridization sites of *Poa*-353 of 8 was probed in tetraploid species with an exceptional number of 20 in *P. paucifolia*, while about 22 sites were detected in the high polyploidy species with an exceptional small number of 5 in *P. subfastigiana* averagely. Roughly. The hybridization sites number of *PpTr-1* and *Poa-*362 is decreasing by the increasing of the polyploidy, but that of *Pp*Tr-3 and Poa-353 is increasing by the increasing of the polyploidy. Specially, *P. pratensis* nearly contains the least number or none of *Poa*-362, and most number of *Poa*-353.

### rDNA distribution patterns

rDNA (45S rDNA and 5S rDNA) are expressed, and highly tandem-repeated in plant genomes. Commonly, the distribution pattern and abundance of rDNA are highly stable in a species. The variable rDNA distribution patterns across different species can provide valuable information to infer the phylogenetic relationship between species. The phylogeny of the *Poa* species used in this study is not clear. It was thought that description of the rDNA patterns across species might be helpful to explain the phylogenetic relationship of the investigated species as well as the satellite DNAs.

Three different FISH patterns of rDNA were uncovered in tetraploid species, while one representative pattern was in high polyploidy species. In tetraploid, most of the species showed four chromosomes containing both 5S rDNA and 45S rDNA each in discrete sites as well as other four chromosomes including only one 45S rDNA site respectively (Fig. 1d2, d3; Table 2). Two tetroploid species *P. paucifolia* and *P. crymophila* showed four chromosomes containing both 5S rDNA and 45S rDNA each in discrete sites as well as other two chromosomes including only one 45S rDNA site respectively (Table 2). One tetroploid species *P. elanata* showed four chromosomes containing both 5S rDNA and 45S rDNA each in discrete sites, two chromosomes containing other more solely 5S rDNA site, and other two chromosomes including only one 45S rDNA site respectively (Fig. 1d4). Identical FISH pattern of rDNA was difficult to obtain due to the variable chromosome number in each high polyploidy species. However, a common feature of FISH pattern of rDNA is distinguished from the most of the tetrploid species, which showed that 2–3 chromosomes shared with 5S rDNA and 45S rDNA sites, 1–4 chromosomes had only 5S rDNA site, and 2–6 chromosome had solely 4SrDNA site (Fig. 1d5, d6; Table 2).

## Discussion

### Evolution of satellite DNAs in *Poa*

Satellite DNAs *Pp*Tr-1, *Pp*Tr-3, *Poa*-362, and *Poa*-535 were detected nearly in all the tested *Poa* species. It suggests that the satellite DNAs identified in *Poa* shared a common satDNA library from a common ancestor. Differential amplification of satellites from this library and acquisition of mutations may lead to interspecific differences in that fraction [27, 28]. Relatively, a rapid sequences divergence rate of satellites has shown to be species-specific [29]. In this study, the highly variable hybridization sites number was observed across different species. The rate of satellite repeat replacement was proposed to occur with the deletion of large chromatin blocks and re-amplification [30]. It implies that detected hybridization sites variation is due to the increasing or decreasing of the repetitive copies number rather than the sequences divergence across the *Poa* species. In addition, the chromosomal distribution of *Pp*Tr-1, *Pp*Tr-3, and *Poa*-362 are stably detected in the subtelomeric regions, but the hybridization number variations between these satellites are not accordingly related. It suggests independent evolving way of the repeats.

The abundance variation of repetitive sequence is following the polyploidy process in plant [31]. In this study, distinct increasing hybridization sites of Pp*Tr-3* and *Poa*-353, and decreasing hybridization sites of *Poa*-362 were observed in the high polyploidy species *P. pratensis*. It proposed the different reaction way of the different satellites to the genome size change.

### Phylogenetic relationship among *Poa* species

*Poa* is large polyploid complex [15] with high degree of poluploids, which are originated by alloypolyploiy or autopolyploidy [15, 32, 33]. *Poa* is with a basic chromosome number of x = 7 [34]. In this study, most of the investigated species are tetraploid species with a chromosome number of 28, with occasionally diploid plants identified in *P. malaca*; two species *P. subfastigiana* and *P. pratensis*, are high polyploidy species with a chromosome number more than 42. From the polyploidy level, these species could be divided into two major groups.

In the tetraploid group, the species can be tentatively separated into three sections by the rDNA FISH patterns., Most of the species in “Background” section, shared a common of rDNA FISH pattern showing a duplicated rDNA pattern revealed in diploid form in *P. malaca*. However, the cytogenetic discrepancy between these species is still apparent, while they were detected by probes *Pp*Tr-1, *Pp*Tr-3, *Poa*-362, and P*oa*-353. Specially, *P. orinosa* was distinct with no hybridization of *Pp*Tr-3. In “Materials and methods” section, one species *P. paucifolia* is far distant from the others with unique rDNA FISH pattern and no hybridizations of *Poa*-535. Two species in “Results” section shared an rDNA FISH pattern different from the others, but *P. paucifolia* is distinguished from P. crymophila by carrying no hybridizations of *Pp*Tr-3. More than 7 maternal genome lineage clades were uncovered in low polyploidy *Poa* species by using chloroplast sequences [35, 36]. It suggests that the diverse genomes may be involved in the tetraploid species in this study. Autopolyplody or alloypolyploidy origins of the species still need further investigation.

In the high polyploidy group, *P. subfastigiana* id distinguished from *P. pratensis* with highly lower hybridization sites of *Poa*-535. *P. pratensis* is a most studied species in genus *Poa*, due to its wide cultivation as forage and turf grass. Genome relationships in *P. pratensis* and other *Poa* species by nuclear sequences revealed four distinct classes of sequences corresponding to 4 putative within polyploidy, and 15 other *Poa* species were found to group with at least 1 *P. pratensis* homoeolog[3330]. Further cytogenetic investigation of the *P. pratensis* related species may provide valuable information to analysis of the genome donors of *P. pratensis*. *P. pratensis* cultivar ‘Qinghai’ and *P. pratensis* Var. *anceps* include much less *PpTr*-3 and more *Poa*-362 hybridization sites than cultivar ‘Park’, ‘Geronimo’ and ‘Sapphire’. Cultivar ‘Qinghai’ and *P. pratensis* Var. *anceps* are nearly wild population collections used in Qinghai, China, while ‘Park’, ‘Geronimo’ and ‘Sapphire’ highly breeding improved by the seed company of USA. Since *P. pratensis* is a facultative apomixes species, the apomictic individuals which can fix the heterosis quickly are usually selected in a breeding scheme [37]. Whether the variation of *PpTr*-3 and *Poa*-362 is related to breeding selection or even to apomictic, it is valuable to elucidate further.

### Efficient exploring satellite DNAs and chromosomal marker in de novo

In the previous study [18], four satellite DNAs were identified by construction and screening of Cot-1DNA libraries by using genomic DNA of *P. prantensis* ‘Qinghai’. Though apparent hybridizations of *Poa*-362 and *Poa*-535 were identified in *P. prantensis *‘Qinghai’ in this study, tandem repeats of those were missing in previous study. It suggests that identification of satellite DNA by RepeatExplorer in de novo is more efficient than traditional approach. Additionally, oligonucleotide desigened probes were used for newly identified *Poa*-362 and *Poa*-535. Prominent hybridizations were produced by these probes. By the decreasing of the NGS cost, it is highly efficient and convenient to identify and characterize satellite repeats by combination of RepeatExplorer and Oligo-FISH.

## Conclusions

Four satellite DNAs were identified by using the RepeatExplorer pipeline to analyse HiSeq Illumina reads in *Poa*. Two showed the high similarity with the previously identified repeats *PpTr*-1 and *PpTr*-3, while two were newly identified (*Poa*-362 and *Poa*-353). By using labelled clone DNA and oligonucleotide designed probes, *Pp*Tr-1, *Pp*Tr-3, and *Poa*-362 were exclusively physically mapped on the subtelomeric regions of the chromosomes in *Poa* species. *Poa-*353 produced hybridization on multiple regions. The variation of hybridization sites number of each satellite was observed in 13 *Poa* species. The tested species could be divided into tetraploidy and high polyploidy species by the chromosome number. In tetraploidy group, *P. elanata* is distant from others with unique rDNA FISH pattern and absence of *Poa*-353 hybridization, as well as *P. paucifolia* is distinct from most species with the different rDNA FISH pattern and absence of *Poa*-Tr3 hybridization. Comparing with tetraploids, high polyploidy species *P. subfatigiana* and *P. prantensis* contained least hybridization sites number of *Poa*-363, and *P. prantensis* involved most number of Poa-353. The usefulness of the satellites for the genome dissection and the phylogeny definition between *Poa* species can be enforced by involving the application of phylogenomic and bioinformatic approaches further.

## Data Availability

The datasets generated and/or analyzed during the current study are not publicly available due individual privacy but are available from the corresponding author on reasonable request.

## Abbreviations

FISH: Fluorescent in situ hybridization
NGS: Next generation sequencing technique
